# Impossible Movement Illusions

**DOI:** 10.1177/2041669518816106

**Published:** 2018-12-20

**Authors:** Richard Wiseman, Will Houstoun

**Affiliations:** University of Hertfordshire, UK; London, UK

**Keywords:** body perception, illusion, magic, motion, perception, perceptual organization

## Abstract

Past research has used the phi phenomenon to create the illusion of one object moving through another. This article presents three optical illusions that are conceptually similar, yet little known within academic psychology. Two of the illusions have been developed within the magic community and involve the performer appearing to make a finger jump from one hand to another and a cup penetrate through another cup. The article explores the factors underpinning these illusions and describes how these factors were used to enhance a similar illusion developed outside of magic (the penetration of one hand through another).

Around the turn of the last century, researchers developed a now classic demonstration of illusory motion known as the ‘phi phenomenon’ (Exner, 1875; Wertheimer, 1912). Observers were first shown an object in one location (e.g., a dot on the left of the visual field) and then, milliseconds later, were shown an identical object in a different location (e.g., a dot on the right of the visual field). When asked to describe what they had seen, observers tended to report seeing the object moving from one location to the other.

More recent research has revealed that this effect is very robust and can even be used to create impossible forms of motion. For example, when shown an image of a ball to the left of a cube, quickly followed by an image of the ball to the right of the cube, observers tend to report seeing the ball penetrate through the cube (Chatterjee, Freyd, & Shiffrar, 1996).

Magicians have developed conceptually similar optical illusions for live performance. Although these illusions are well documented within the magic community, they are little known within academic psychology. Perhaps the simplest of these involves the magician extending the first finger of their right hand and placing their hands side by side. After moving the hands apart and together a few times, the magician rapidly retracts the first finger of their right hand and simultaneously extends the first finger of their left hand. Properly performed, the magician’s finger appears to have jumped from one hand to another (for a video depicting how this effect could look in performance, see Movie 1).

**Movie 1.** Finger Illusion.

Similarly, during the classic ‘Cups and Balls’ trick, some magicians hold one of the cups mouth up in one hand and a second cup mouth up in their other hand. The magician then places one hand above the other and drops the upper cup. As the top cup falls into the lower cup, the magician releases the lower cup and holds onto the top cup. Properly timed, the top cup appears to penetrate through the lower cup (Movie 2). This optical illusion was developed a considerable time ago, with written records dating back to the late the 18th century (Decremps, 1788).

**Movie 2.** Cup Illusion.

Another example of these ‘impossible movement’ optical illusions has recently appeared in several online videos and involves one hand appearing to move through the other (Evans, 2018). During this illusion, the performer extends the fingers of both hands and places their right hand behind their left hand. They then bend the fingers of their right hand and thread them through the gaps between the fingers of their left hand. Quickly extending the fingers of their right hand and simultaneously closing the fingers of the left hand gives rise to the illusion that one hand has penetrated through the other (Movie 3).

**Movie 3.** Our version of the Hand Illusion.

The success of these types of illusions appears to depend on two main factors: ‘similarity’ and ‘trajectory’. It is clearly important that the object seen prior to the impossible movement resembles the object seen after the movement. In the Finger Illusion, the magician’s left first finger is almost identical to their right first finger. Likewise, the Cup Illusion involves exchanging two identical objects and in the Hand Illusion the magician’s right hand looks the same as left hand. Second, both illusions involve a strong sense of an anticipated trajectory. In the Finger Illusion, the magician repeatedly knocks their hands together before their finger apparently jumps from one hand to the other, in the Hand Illusion the magician’s right hand moves towards their left hand, and during the Cup Illusion both cups move along the same trajectory. In this sense, the illusions are reminiscent of the important role that trajectory plays in creating a sense of object permanence (see, e.g., Spelke, 1990) and reflect key ideas within Gestalt psychology on grouping (namely, ‘similarity’ and ‘Good Continuity’).

Using these principles, we have created two modified versions of the Hand Illusion. In one, the right hand repeatedly approaches the left hand before the penetration, thus creating an enhanced sense of trajectory (Movie 4). In the other one, the fingers of the right hand are additionally bent over and straightened prior to the penetration. Then, one moment before the penetration, the left hand adopted the same bend positioning. This is designed to create a stronger sense of similarity between the two hands (Movie 5).

**Movie 4.** The hand illusion with improved continuity.

**Movie 5.** The hand illusion with improved similarity.

These somewhat unusual optical illusions are not well known within psychology. It is hoped that they may prove helpful within both research and teaching, and add to the growing list of illusions that have moved out of the magician’s repertoire and into the laboratory (Kuhn, Olson, & Raz, 2016).
